# Targeting on Asymmetric Dimethylarginine-Related Nitric Oxide-Reactive Oxygen Species Imbalance to Reprogram the Development of Hypertension

**DOI:** 10.3390/ijms17122020

**Published:** 2016-12-02

**Authors:** You-Lin Tain, Chien-Ning Hsu

**Affiliations:** 1 Departments of Pediatrics, College of Medicine, Kaohsiung Chang Gung Memorial Hospital and Chang Gung University, Kaohsiung 833, Taiwan; tainyl@hotmail.com; 2 Institute for Translational Research in Biomedicine, College of Medicine, Kaohsiung Chang Gung Memorial Hospital and Chang Gung University, Kaohsiung 833, Taiwan; 3 Department of Pharmacy, Kaohsiung Chang Gung Memorial Hospital, Kaohsiung 833, Taiwan; 4 School of Pharmacy, Kaohsiung Medical University, Kaohsiung 807, Taiwan

**Keywords:** asymmetric dimethylarginine, dimethylarginine dimethylaminohydrolase, hypertension, nitric oxide, oxidative stress

## Abstract

Adult-onset diseases, including hypertension, can originate from early life, known as the developmental origins of health and disease (DOHaD). Because the developing kidney is vulnerable to early-life insults, renal programming is considered key in the developmental programming of hypertension. Asymmetric dimethylarginine (ADMA), an endogenous nitric oxide (NO) synthase inhibitor, can regulate the NO–reactive oxygen species (ROS) balance, and is involved in the development of hypertension. Reprogramming interventions aimed at NO-ROS balance can be protective in both genetic and developmentally programmed hypertension. Here we review several emergent themes of the DOHaD approach regarding the impact of ADMA-related NO-ROS imbalance on programmed hypertension. We focus on the kidney in the following areas: mechanistic insights to interpret programmed hypertension; the impact of ADMA-related NO-ROS imbalance in both genetic and acquired animal models of hypertension; alterations of the renal transcriptome in response to ADMA in the developing kidney; and reprogramming strategies targeting ADMA-related NO-ROS balance to prevent programmed hypertension.

## 1. Introduction

Hypertension remains an important public health challenge, despite progress made in recent years in antihypertensive therapies. Early interventions to prevent the onset of hypertension are, therefore, solutions for the future to save lives and impact even greater health care savings. Hypertension might originate during fetal development and early childhood [1]. The concept that adult-onset diseases have an early-life origin has been raised, referred to as the developmental origins of health and disease (DOHaD) [2]. Early-life redox imbalance may lead to permanent alterations of function and structure in later life in specific organs that are vulnerable to developing hypertension [3,4]. Asymmetric dimethylarginine (ADMA), an endogenous nitric oxide (NO) synthase inhibitor, can inhibit nitric oxide synthase (NOS) and regulate the NO–reactive oxygen species (ROS) balance [5]. Cumulative evidence implicates the role of ADMA-induced NO-ROS imbalance in the developmental programming of hypertension [6,7].

As the developing kidney is particularly vulnerable to early-life insults, renal programming has been linked to programmed hypertension [8]. The DOHaD concept also offers a novel approach to preventing hypertension and related cardiovascular diseases, through so-called reprogramming [7]. Reprogramming strategies have been proposed for restoring the NO-ROS imbalance to prevent the development of hypertension [9,10].

This review aims to summarize evidence linking ADMA-related NO-ROS imbalance to the development of hypertension with a focus on the kidney and to provide various manipulations of the ADMA-NO pathway prior to hypertension in favor of NO as a reprogramming approach to prevent programmed hypertension.

## 2. Developmental Origins of Hypertension: Focus on the Kidney

The kidney is a major organ in long-term blood pressure (BP) regulation. As the developing kidney is vulnerable to environmental insults, the kidney has therefore been considered as a leading player in the development of hypertension [6,8,11]. Numerous epidemiologic observations support that prematurity and low birth weight are risk factors for subsequent hypertension in later life, which may be mediated by reduced nephron endowment [1,11,12,13]. So far, however, the amount of nephron cannot be determined in vivo. Some hypotheses related to the DOHaD concept have been proposed in attempts to better explain these epidemiological observations, such as the thrifty phenotype [14], programming and predictive adaptive response theories [15], and the catch-up growth hypothesis [16]. Nevertheless, these hypotheses cannot suggest possible molecular mechanisms whereby the phenotype is generated. Early-life insults cause structural and functional changes in the developing kidney by so-called renal programming [11]. It stands to reason that much of our knowledge of the types of insults, the period of vulnerability for insults, and the potential mechanisms involved in renal programming has been acquired using animal research.

**Figure 1:**
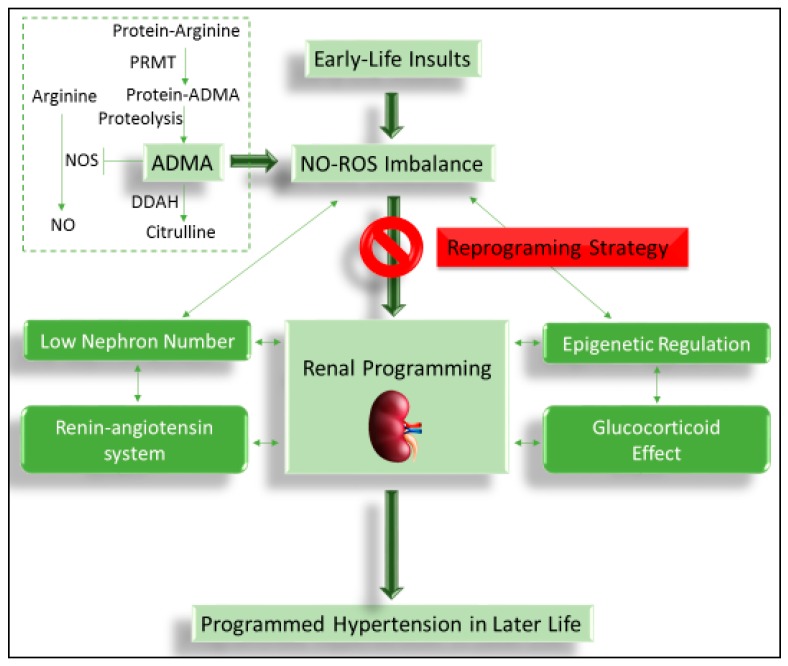
Schema outlining the central role of NO-ROS imbalance on mediating other mechanisms in the kidney, leading to programmed hypertension in response to early-life insults. Targeting the ADMA-related NO-ROS imbalance could be a therapeutic option to prevent the development of programmed hypertension to reduce the lifetime burden with early intervention.

A large number of animal studies have documented the association between suboptimal conditions in the prenatal period, renal programming, and subsequent programmed hypertension in adult offspring [8,9,11]. Although our understanding of the common mechanisms underlying the programming of hypertension remains far from complete, experimental models have provided certain hypothetical mechanisms, including but not limited to epigenetic regulation, glucocorticoid effects, inappropriate suppression or activation of the renin-angiotensin system (RAS), low nephron number, and NO-ROS imbalance [7,8,11]. Importantly, among these proposed mechanisms, NO-ROS imbalance is closely inter-related to the others in determining the programmed hypertension process (Figure 1).

Four lines of evidence indicate that the NO-ROS imbalance interacts with other mechanisms to provoke programmed hypertension. First, a growing body of evidence indicates that redox signaling plays a pivotal role in epigenetic regulation, including the methylation of CpG islands, and the modification of histone proteins and microRNAs [17]. A disturbed balance between NO and ROS is involved in the epigenetic regulation of genes implicated in control of BP in a variety of programmed hypertension models [18,19,20]; Second are studies of oxidative stress on glucocorticoid-induced hypertension [21]. The NO-ROS imbalance has been reported in several models of glucocorticoid-induced programmed hypertension [20,22,23,24,25,26,27]; Third are many reports that angiotensin II–induced oxidative stress plays an important role in the development of hypertension [6,28,29], whereas early blockade of the RAS has been shown to deprogram the inappropriately activated RAS and reduce oxidative stress to prevent the development of programmed hypertension [30,31]; Last, epidemiologic studies support that low birth weight and prematurity, both associated with reduced nephron endowment, are risk factors for hypertension in later life [32,33]. A reduced nephron number could cause higher glomerular capillary pressure and glomerular hyperfiltration, consequently initiating a vicious cycle of renal damage and nephron loss leading to rising BP. Fewer nephron numbers were found in patients with primary hypertension [34]. Multiple animal models of programmed hypertension have demonstrated the association of NO-ROS imbalance with a decline in the nephron number [8,23,35,36]. All of these observations provide a close link between the NO-ROS imbalance and other important mechanisms involved in programmed hypertension.

## 3. ADMA-Induced NO-ROS Imbalance in Programmed Hypertension

Emerging evidence demonstrates that ADMA is involved in the development of hypertension and related cardiovascular diseases [5,37,38]. ADMA is a natural amino acid, which can inhibit the activity of NOS to reduce NO production (Figure 1). Protein-incorporated ADMA is a methylated arginine derivative generated by the addition of methyl groups in arginine residue in proteins through the type I protein arginine methyltransferase (PRMT) family. Free ADMA is then released following proteolysis [5,38]. Two other derivatives methylated by PRMTs are symmetric dimethylarginine (SDMA) and monomethylarginine (MMA). Free ADMA can be transported to other organs by cationic amino acid transporter (CAT) or excreted by urine. Approximately 80% of ADMA is metabolized by dimethylarginine dimethylaminohydrolase-1 (DDAH-1) or -2 (DDAH-2), mainly in the kidneys, liver, and endothelium [5,38].

ROS have been shown to increase PRMT and inhibit DDAH activity, leading to an increase in ADMA [39,40]. On the other hand, the NOS isoenzymes become uncoupled to produce peroxynitrite in the presence of high ADMA levels, further contributing to the burden of oxidative stress [41]. Thus, ADMA may contribute to the production of ROS and reactive nitrogen species (RNS). The overview of studies in Table 1 illustrates data documenting ADMA-related NO-ROS imbalance in both genetic and acquired animal models of hypertension.

## 4. The Impact of ADMA-Induced NO-ROS Imbalance on Developing Kidney

So far, very few studies have addressed genomic and transcriptomic research to explore and identify common biological traits in a set of genes in the kidney in response to a variety of early-life insults [50,51,52,53]. However, no studies of the developing kidney have been reported regarding the NO-ROS imbalance as important for the programming of hypertension. Because renal programming plays a crucial role in programmed hypertension [8], we used to analyze the renal transcriptome in adult offspring kidney using next-generation RNA sequencing (NGS) technology in three programmed hypertension models, including a model of NO inhibition by *N^G^*-nitro-l-arginine-methyester (l-NAME) [50]. Among them, a total of five shared differential expressed genes (DEGs), *Bcl6*, *Dmrtc1c*, *Egr1*, *Inmt*, and *Olr1668*, were identified among three different models. In the l-NAME model, there was a total of 383 DEGs (198 up- and 185 down-regulated genes by l-NAME vs. control). In addition, we found five out of the 383 DEGs, namely *Apln*, *Guca2b*, *Hmox1*, *Hba-a2*, and *Npy*, were related to the regulation of BP (GO: 0008217). Moreover, NO depletion in pregnancy induced by l-NAME caused a wide range of signaling pathways as found by Kyoto Encyclopedia of Genes and Genomes (KEGG) pathway analysis. The top nine related KEGG pathways that are significantly overrepresented include the mitogen-activated protein kinases (MAPK) signaling pathway, the circadian rhythm, colorectal cancer pathways , the NOD-like receptor signaling pathway, renal cell carcinoma, the Wnt signaling pathway, prion diseases, and the chemokine signaling pathway [50]. Whether these genes are related to NO-ROS imbalance in response to l-NAME leading to programmed hypertension, and in particular whether they are potential target genes and pathways for reprogramming interventions, awaits further elucidation.

Compared to the adult kidney, the developing kidney in the fetus might be the critical window for programming susceptibility to identify candidate genes and pathways associated with the development of hypertension. Hence, analyzing the transcriptome in embryonic kidneys (metanephroi) offers a potential solution to identify early-life insult-induced primary programmed changes.

We used to evaluate whether ADMA can impair nephrogenesis [36]. Metanephroi from fetuses at embryonic day 14 (E14) were collected, treated with different concentrations of ADMA (2 and 10 µM), and harvested after six days. Metanephroi grown in 2 or 10 µM ADMA were significantly smaller and contained fewer nephrons in a dose-dependent manner. We next analyzed the renal transcriptome in response to ADMA in the developing kidney using the NGS approach. Metanephroi grown in 10 µM ADMA (*n* = 3/group) were isolated for NGS analysis, performed by Welgene Biotech Co., Ltd. (Taipei, Taiwan), as we described previously [50,53]. A total of 1221 DEGs (735 up- and 486 down-regulated genes by ADMA vs. control) met the selection criteria of (1) genes that changed by reads per kilobase of transcript per million mapped reads (RPKM) >0.3 and (2) a minimum 1.5-fold difference in normalized read counts between groups.

For functional annotation and biological mechanism analyses, the DAVID v6.7 [54] bioinformatics tool was used. Also, we identifies specifically enriched Gene Ontology (GO) groups to explore distinct gene networks involved in ADMA-related programmed hypertension. As shown in Table 2, we found that eight of the 1221 ADMA-induced DEGs, namely *Avpr1a*, *Chrna7*, *Ephx2*, *Hba2*, *Hba-a2*, *Npy1r*, *P2rx2*, and *Tnni3*, were related to the regulation of BP (GO: 0008217). Among them, *Avpr1a*, *Ephx2*, *Hba2*, *Hba-a2*, and *Npy1r* have been identified as differentially expressed genes in the kidney in a variety of programmed hypertension models [25,50,53,55]. Soluble epoxide hydrolase (SEH) is an enzyme that is encoded by the *Ephx2* gene. SEH hydrolyzes epoxyeicosatrienoic acids (EETs) to dihydroxyeicosatrienoic acids (DHETs). EETs cause vasodilation whereas DHETs cause vasoconstriction [56]. Upon SEH inhibition, EETs accumulate and provoke vasodilation to lower BP. It is noteworthy that *Ephx2* gene expression and SEH activity seem to play a crucial role in a variety of programmed hypertension models [25,50,55]. On the other hand, SEH inhibitors could lower BP in several animal models of hypertension [56]. Indeed, we recently reported that early inhibition of SEH with the orally active inhibitor 12-(3-adamantan-1-yl-ureido)-dodecanoic acid (AUDA) could prevent programmed hypertension in the dexamethasone and high fructose models [26,57]. Our observations suggest that there might be common pathways by which different early-life insults elicit the same phenotype in the adult offspring–programmed hypertension.

Given that NO regulates many physiological functions, it is not surprising that several important biological pathways are regulated by ADMA in the developing kidney during nephrogenesis. There were 13 significantly related KEGG pathways in the developing kidney treated with ADMA (Table 3). Among them, the chemokine signaling pathway, the NOD-like receptor signaling pathway, and the MAPK pathway have been identified in the l-NAME-induced programmed hypertension model [58]. The MAPK pathway is involved in redox-sensitive signaling, contributing to the development of hypertension [59]. The arachidonic acid metabolism pathway is also a significant related KEGG pathway. It is noteworthy that our recent reports show that arachidonic acid metabolites can program hypertension with different insults, such as prenatal dexamethasone exposure and maternal high fructose consumption [25,55]. Our data suggest that the arachidonic acid metabolism pathway might be a common pathway contributing to programmed hypertension in diverse animal models. Furthermore, our data showing that ribosome is a significant KEGG pathway support a previous report showing that perinatal NO administration alters renal ribosome biogenesis in a genetically hypertensive rat model [60].

## 5. Reprogramming Strategy via Targeting ADMA to Restore NO-ROS Balance

Oxidative stress has been considered as a major mechanism contributing to programmed hypertension [8,9]. It would be logical to consider antioxidant supplementation in potential therapies for hypertension and related cardiovascular diseases. However, so far, antioxidant therapy is not proving to be a panacea to control the global rise of hypertension [61]. A number of recent studies addressed reprogramming interventions aimed at the restoration of the NO-ROS balance, such as perinatal supplements of citrulline, melatonin, Vitamin C or E, which can be protective in programmed hypertension; this has been reviewed recently [9]. Although ADMA is considered as a major player leading to NO-ROS imbalance, little attention has been paid to targeting the restoration of ADMA-induced NO-ROS imbalance to prevent programmed hypertension.

So far, a specific ADMA-lowering agent remains inaccessible. As we previously reviewed, a number of medications have been reported to lower ADMA levels in human studies, including angiotensin-converting enzyme inhibitors, angiotensin receptor blockers, fenofibrate, folic acid, metformin, oral contraceptives, and α-lipoic acid [9]. As we mentioned earlier, PRMTs control ADMA production whereas DDAHs regulate its metabolism. Hence, the discovery of specific PRMT inhibitors or DDAH agonists might become a useful approach. However, the creation of specific PRMT inhibitors remains challenging because of the high degree of sequence conservation across the PRMT family throughout evolution [62]. On the other hand, regulation of DDAH enzymes might lead to a therapeutic target to treat ADMA-induced NO-ROS imbalance. A number of animal studies have indicated that pravastatin, aminoguanidine, pioglitazone, probucol, farnesoid X receptor agonists, vitamin E, melatonin, resveratrol, *N*-acetylcysteine, and aliskiren can increase the activity and/or expression of DDAH, and thereby reduce ADMA levels [9,30,47,63]. Over the past two decades, an increasing number of studies have revealed that ADMA is a cardiovascular risk factor, a diagnostic marker for a broad variety of diseases, and a potential therapeutic target [5,10,37]. Additional studies exploring its role as a target for drug development in the prevention and treatment of hypertension are highly warranted.

## 6. Conclusions

Patients with prehypertension have an increased risk of full-blown hypertension and cardiovascular-related morbidity and mortality [64]. Increasing evidence, including epidemiological observations and experimental animal studies, demonstrates that early-life ADMA-related NO-ROS imbalance may lead to permanent alterations of function and structure in later life in the kidney to develop programmed hypertension in adult life. In contrast, reprogramming interventions aimed at shifting the redox balance can be protective in both genetic and developmentally programmed hypertension. Thus, early detection of individuals that are at risk for hypertension and early reprogramming interventions targeting the ADMA-related NO-ROS imbalance to prevent the development of programmed hypertension can be translated into clinical practice and will allow us to reduce the globally growing epidemic of hypertension and related cardiovascular diseases.

## Figures and Tables

**Table 1:** ADMA related NO-ROS imbalance in programmed hypertension models.

Programming Mechanism	% ADMA Increase from Controls	Programming Effects	Age at Which Effects Were Measured	Reference
Spontaneously hypertensive rat (SHR)	↑ 51%	Hypertension	Until 10 weeks of age	[30]
SHR treated with l-NAME from 4 to 10 weeks of age	↑ 50%	Hypertension, renal injury	Until 10 weeks of age	[42]
Dahl salt-sensitive rat treated with high salt	↑ 23%	Hypertension	Until 11 weeks of age	[43]
SHR	↑ 34%–51%	Hypertension	Until 12 weeks of age	[44,45,46,47,48]
50% caloric restriction during pregnancy and lactation	↑ 69%	Hypertension	Until 12 weeks of age	[19,31]
Diabetes (STZ) during pregnancy and lactation	↑ 579% (in the kidney)	Hypertension, renal injury	Until 12 weeks of age	[36]
Suramin during pregnancy	↑ 52%	Hypertension	Until 12 weeks of age	[49]
Dexamethasone during pregnancy	↑ 22%	Hypertension	Until 16 weeks of age	[24]

Studies tabulated according to age at which effects were measured.

**Table 2:** Changes in genes related to regulation of BP in the developing kidney treated with ADMA versus control.

Gene ID	Gene Symbol	Description	Fold Changes ADMA/Control
ENSRNOG00000004400	*Avpr1a*	arginine vasopressin receptor 1A	0.47
ENSRNOG00000010853	*Chrna7*	cholinergic receptor, nicotinic, α 7	0.57
ENSRNOG00000014149	*Npy1r*	neuropeptide Y receptor Y1	1.65
ENSRNOG00000017286	*Ephx2*	epoxide hydrolase 2, cytoplasmic	1.68
ENSRNOG00000018250	*Tnni3*	troponin I type 3 (cardiac)	3.97
ENSRNOG00000031686	*Hba2*	hemoglobin α 2 chain	2
ENSRNOG00000029886	*Hba-a2*	hemoglobin α, adult chain 2	0.63
ENSRNOG00000037456	*P2rx2*	purinergic receptor P2X, ligand-gated ion channel 2	1.88

**Table 3:** Significantly regulated KEGG pathways in the developing kidney treated with ADMA versus control.

Term	Count	%	*p*-Value	Benjamini
Ribosome	12	1.1	2.4 × 10^−^^4^	3.2 × 10^−^^2^
Cytokine-cytokine receptor interaction	19	1.8	3.9 × 10^−^^4^	2.6 × 10^−^^2^
Chemokine signaling pathway	16	1.5	1.8 × 10^−^^3^	7.7 × 10^−^^2^
Neuroactive ligand-receptor interaction	21	2.0	1.8 × 10^−^^3^	6.0 × 10^−^^2^
Arachidonic acid metabolism	9	0.8	4.4 × 10^−^^3^	1.1 × 10^−^^1^
Intestinal immune network for IgA production	7	0.7	6.3 × 10^−^^3^	1.3 × 10^−^^1^
Systemic lupus erythematosus	10	0.9	6.3 × 10^−^^3^	1.1 × 10^−^^1^
Toll-like receptor signaling pathway	9	0.8	1.9 × 10^−^^2^	2.8 × 10^−^^1^
NOD-like receptor signaling pathway	7	0.7	2.8 × 10^−^^2^	3.4 × 10^−^^1^
Tyrosine metabolism	5	0.5	3.7 × 10^−^^2^	4.0 × 10^−^^1^
MAPK signaling pathway	17	1.6	4.1 × 10^−^^2^	4.0 × 10^−^^1^
Cell adhesion molecules (CAMs)	11	1.0	5.1 × 10^−^^2^	4.4 × 10^−^^1^
Vascular smooth muscle contraction	9	0.8	6.1 × 10^−^^2^	4.8 × 10^−^^1^

## Abbreviations

ADMA	Asymmetric dimethylarginine
AUDA	12-(3-Adamantan-1-yl-ureido)-dodecanoic acid
CAT	Cationic amino acid transporter
DDAH	Dimethylarginine dimethylaminohydrolase
DEG	Differential expressed gene
DOHaD	Developmental origins of health and disease
KEGG	Kyoto Encyclopedia of Genes and Genomes
l-NAME	*N^G^*-nitro-l-arginine-methyester
MAPK	Mitogen-activated protein kinases
NGS	Next generation RNA sequencing
NOS	Nitric oxide synthase
PRMT	Protein arginine methyltransferase
RAS	Renin-angiotensin system
RPKM	Reads per kilobase of transcript per million mapped reads
SDMA	Symmetric dimethylarginine
